# Alpha Therapy Beyond TOC and TATE—Production, Quality Control, and In-Human Results for the SSTR2 Antagonist DOTA-LM3

**DOI:** 10.3390/ph19010172

**Published:** 2026-01-19

**Authors:** Lukas Greifenstein, Marcel Martin, Sarah Stephan, Aleksandr Eismant, Carsten S. Kramer, Christian Landvogt, Corinna Mueller, Frank Rösch, Richard P. Baum

**Affiliations:** 1 CURANOSTICUM MVZ GmbH Wiesbaden-Frankfurt, Center for Advanced Radiomolecular Precision Oncology, 65191 Wiesbaden, Germany; 2 Department of Chemistry—TRIGA, Institute of Nuclear Chemistry, Johannes Gutenberg University, 55122 Mainz, Germany

**Keywords:** targeted alpha therapy (TAT), Actinium-225 (Ac-225), peptide receptor radionuclide therapy (PRRT), somatostatin receptor 2 (SSTR2) antagonist, neuroendocrine tumors (NETs), radiopharmaceuticals, DOTA-LM3, radionuclide therapy, theranostics, molecular imaging

## Abstract

**Objectives**: Peptide receptor radionuclide therapy (PRRT) of neuroendocrine tumors (NETs) commonly relies on somatostatin receptor subtype 2 (SSTR2) agonists such as DOTA-TOC/TATE, which may show limited efficacy due to high hepatic uptake and therapy resistance in some patients. SSTR2 antagonists have demonstrated superior tumor targeting. This study aimed to establish the production and quality control of the Actinium-225-labeled SSTR2 antagonist [^225^Ac]Ac-DOTA-LM3 and to report in-human clinical experience with targeted alpha therapy (TAT). **Methods**: [^225^Ac]Ac-DOTA-LM3 was produced by radiolabeling DOTA-LM3 with Actinium-225 under validated conditions. Radiochemical conversion, purity, yield, and stability were assessed using radio-TLC, fractionated radio-HPLC combined with gamma spectroscopy, and in vitro serum stability testing. Clinical feasibility and therapeutic response were evaluated in a patient with metastatic neuroendocrine pancreatic neoplasm refractory to prior ^177^Lu-based PRRT. **Results**: Radiolabeling achieved reproducibly high radiochemical purity (>97%) and decay-corrected yields exceeding 80%. The radiopharmaceutical showed high in vitro stability with minimal release of free Actinium-225 over five days. Fractionated radio-HPLC enabled indirect purity assessment. In the reported patient, [^225^Ac]Ac-DOTA-LM3 therapy resulted in partial remission without clinically relevant hematologic, renal, or hepatic toxicity and was associated with marked clinical improvement. **Conclusions**: [^225^Ac]Ac-DOTA-LM3 can be produced with high purity and stability using clinically applicable procedures. In-human results suggest promising efficacy and safety, supporting further clinical investigation of Actinium-225-labeled SSTR2 antagonists for advanced NETs.

## 1. Introduction

**Figure 1:**
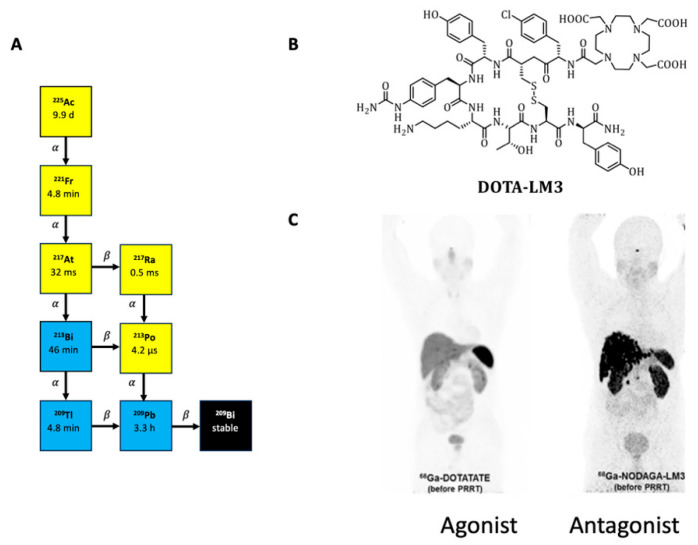
Overview of targeted alpha therapy using Actinium-225-labeled somatostatin receptor antagonists: (**A**) Structure of [^225^Ac]Ac-DOTA-LM3. (**B**) Schematic representation of Actinium-225 decay with emitted particles. (**C**) Comparison of the biodistribution profiles of SSTR2 agonists and antagonists in PET (maximum intensity projections, [^68^Ga]Ga-DOTA-TATE vs. [^68^Ga]Ga-NODAGA-LM3) (image partially adapted from [25]).

Actinium-225 is a radioactive isotope that has gained significant attention in the field of nuclear medicine due to its potential applications in imaging and therapy [1,2]. Actinium-225 is an alpha emitter with a half-life of 9.9 days, emitting four high-energy alpha particles that have a limited range but high biological effectiveness [3] (Figure 1B). Alpha particles are helium-4 nuclei consisting of two protons and two neutrons, giving them an overall charge of +2. This double positive charge, in combination with their weight, contributes to their high linear energy transfer (LET), ranging from 50 to 230 keV/μm. Therefore, alpha particles more often induce DNA double-strand breaks, resulting in a higher target cell toxicity compared to their β-particle counterparts with lower LET [4,5,6]. Additionally, the typical ranges of α-particles in tissue are approximately 100 μm, which is significantly shorter than the millimeter-scale range of β-particles. The combination of these features makes therapy with alpha emitters extremely attractive for radioligand therapy (RLT). So far, only a few radiopharmaceutical approaches have been conducted with small-molecule-based or peptide-based radiopharmaceuticals for targeted alpha therapy (TAT) with Ac-225, i.e., [^225^Ac]Ac-DOTA-TOC/TATE (DOTA: 1,4,7,10-tetraazacyclododecane-1,4,7,10-tetraacetic acid) for treatment of neuroendocrine tumors (NETs); [^225^Ac]Ac-PSMA-617/I&T (PSMA: prostate specific membrane antigen) for the treatment of metastasized prostate cancer; and ^225^Ac-labeled fibroblast activation protein (FAP)-targeting ligands for the treatment of various malignancies [7,8,9,10,11]. Furthermore, TAT was conducted successfully with other alpha emitters such as Pb-212 [12,13,14,15], Th-227 [16], and At-211 [17].

Remarkably, numerous patient cases were reported where standard RLT with the beta-emitter Lutetium-177 failed to generate a treatment response. However, under TAT, greater responses were observed, particularly in the treatment of neuroendocrine tumors [18,19].

NETs are a heterogeneous group of tumors primarily found in the gastrointestinal tract, pancreas, and lungs. The overexpression of somatostatin receptor 2 (SSTR2) in these tumors makes it a target for the diagnosis and treatment of these malignancies [20]. Currently, SSTR2-targeted imaging and peptide receptor radionuclide therapy (PRRT) mainly utilize SSTR2 agonists such as DOTA-TOC/TATE or related molecules. Despite their overall high sensitivity and specificity in SSTR2 imaging, the high hepatic tracer uptake of these compounds poses a challenge in identifying smaller liver metastases. In terms of therapy, some patients do not respond adequately to treatment with agonists [21]. To address these limitations, a new class of SSTR2-agents, specifically SSTR2 antagonists, is garnering significant interest [22,23,24,25,26]. These antagonists have shown promising properties, including increased tumor uptake, improved tumor-to-background ratio, and enhanced tumor retention, for example, in the form of DOTA-LM3 [18], JR-11 [27], and the recently published ligand DATA-LM4 [28]. Labeling of the antagonists with Gallium-68 improves significantly the tumor visualization via positron emission tomography (PET), compared to the agonistic counterparts (Figure 1C), while therapeutic nuclides, such as Lutetium-177 or the emerging Auger emitter Terbium-161, are complexed for therapeutic usage [29]. Remarkably, especially with [^177^Lu]Lu-DOTA-LM3, significant response rates were observed [25].

In the context of TAT, Actinium-225-labeled SSTR2-agonists such as [^225^Ac]Ac-DOTA-TOC or [^225^Ac]Ac-DOTA-TATE have already been described [21,30,31,32]. Building on the success of therapy with Actinium-225-labeled agonists and supported by the already acquired data on the antagonistic ligand [^177^Lu]Lu-DOTA-LM3, we aimed to investigate the synergistic potential of combining both advantages in the radioligand [^225^Ac]Ac-DOTA-LM3 for targeted alpha-particle therapy of NETs.

However, the development and application of Actinium-225 radiopharmaceuticals come with their own challenges. These include the presence of radioactive decay products, more complex quality control, the need for stringent radiation safety measures, potential side effects, accurate dosimetry, and considerations of availability and production costs. Consequently, addressing these challenges is crucial to ensure the safe and effective use of Actinium-225-labeled radiopharmaceuticals in clinical practice.

In this work, we aim to provide a comprehensive overview of our work with [^225^Ac]Ac-DOTA-LM3, putting special focus on the importance of quality control. By highlighting these aspects, we hope to contribute to the advancement of Actinium-225-based therapies and enhance the understanding of their potential in the management of NETs and other malignancies.

## 2. Results and Discussion

### 2.1. Radiochemistry

**Figure 2:**
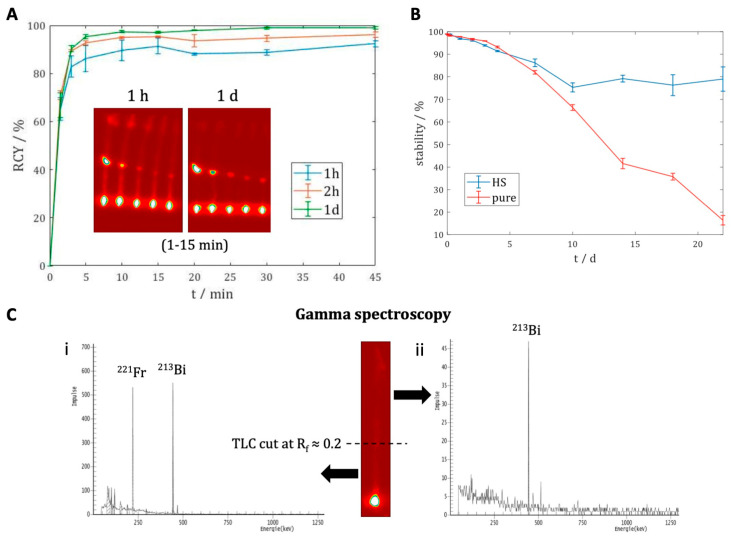
Radiochemical investigation of [^225^Ac]Ac-DOTA-LM3: (**A**) Kinetic investigation of the labeling process (each at 1, 3, 5, 10, 15, 20, 30, and 45 min during labeling) at different decay time points of the plate after development with lines in blue (1 h), orange (2 h) and green (1 d); the images represent the kinetic investigations between 1 and 15 min either 1 h and 1 d after development. (**B**) Stability analysis of [^225^Ac]Ac-DOTA-LM3: unprocessed sample (red line) and as an aliquot in human serum (HS, blue line) at 37 °C. Activity concentrations for the pure solution were up to 1 MBq/mL, and for the HS, 100 kBq/mL. The radiopharmaceutical remained stable for up to five days, with minimal release of free Actinium-225. (**C**) Gamma spectroscopy of the developed TLC plate after development (i) and with a waiting period of 24 h (ii). The lower half of both plates shows energies of Fr-221 and Bi-213, the upper half only shows Bi-213, indicating close to no unbound Ac-225 in the upper part, as no Fr-211 can be observed in the spectrum. All TLCs are developed in citric acid buffer. The arrows indicate which spectrum belongs to which part of the TLC plate, the dotted line indicates the cutting line.

Radiolabeling of DOTA-LM3 with Actinium-225 was performed in a manual process in a shielded glove box in sodium ascorbate buffer at 95 °C for 45 min using 25 µg of DOTA-LM3/MBq [^225^Ac]AcCl_3_. We used no further purifications for Ac-225 before or after the synthesis, and formulations were carried out in a solution of 9 mL saline for injection with sodium ascorbate and ethanol. For robust production, the above-mentioned parameters were used for process validation (*n* = 3). The focus during validation was placed on the following specifications: a radiochemical conversion (RCC) of >95%, which was assessed by radio thin-layer chromatography (TLC) analyzed at different time points after development (1 h, 2 h, and 24 h; see Figure 2A) and gamma spectroscopy analyzing the share of the Fr-221 and Bi-213 daughter lines of the Ac-225 (see Figure 2C: left and right after development of the plate; right, 1d, after development). These measurements, especially the radio-TLC, are usually easy to obtain and deliver fast results after only 1 h of waiting time after development. Additionally, our focus was to establish a reliable assessment of the approximation of radiochemical purity (RCP) using a method other than radio-TLC, given access to information on the real purity of the compound, and to enable inferences on potential decomposition of the molecule. It is necessary to note that this still only represents an indirect measurement and therefore an approximation. These measurements do not align with the regulatory need for real pharmaceutical purities, as we are not capable of separating the products labeled with unavoidable progeny isotopes. When speaking of RCP throughout this work, we always intend to mean it in the sense of the sum of all products that can be produced by Ac-225 and its progeny. We additionally do not consider them dangerous impurities but rather a benefit. This means that whenever we refer to the RCP of [^225^Ac]Ac-DOTA-LM3, [^213^Bi]Bi-DOTA-LM3 is also measured and included in the RCP. This also applies to unbound Bi-213 when measuring unbound activity.

Therefore, we used RCP assessment by radio-high-performance liquid chromatography (HPLC) and subsequent fractioning and gamma spectroscopy. Furthermore, due to the high cost of Ac-225, we included a radiochemical yield (RCY) of >80% to the set parameters.

**Figure 3:**
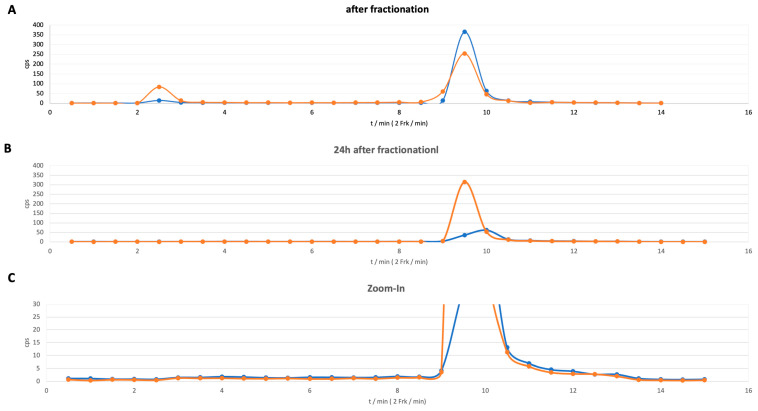
Radio-HPLC analysis from fractionated gamma spectroscopy of [^225^Ac]Ac-DOTA-LM3; orange: Fr-211, blue: Bi-213: (**A**) Chromatogram immediately after separation. (**B**) Gamma spectroscopy results after 24 h confirm the absence of Ac-225 in the first signal. (**C**) Zoom-in of chromatogram B to resolve the signal of a typical uncharacterized impurity at 12.5 min. Conditions for HPLC: column: VDSpher PUR 150 C18-E 5 μm, 100 × 4.0 mm; mobile phases acetonitrile (**A**) and water (**B**), each containing 0.1% TFA; gradient with a flow rate of 1.0 mL/min from 5% solvent A to 95% of A within 10 min. Fraction volumes: 0.5 mL, resulting in a total of 16 vials.

Therefore, a fractionated radio-HPLC was performed, in which the sample was separated by HPLC and subsequently collected in 0.5 mL aliquots. This approach was chosen because (i) only relatively small amounts of activity are applied to the HPLC, resulting in very low counting yields for gamma emission in conventional detectors; (ii) direct detection of alpha particles requires scintillation liquids and specific detectors that are not readily available in every laboratory; and (iii) fraction collection provides valuable information on the origin of the different isotopes eluting from the column. Since Ac-225 solutions are purified by the supplier only up to 14 days prior to clinical application, the formation of decay products is inevitable. Consequently, daughter nuclides such as Bi-213 and, even more prominently, Fr-221 may appear at shorter retention times, reflecting unbound activity in the chromatogram (see Figure 3A). Reevaluating the collected fractions after 24 h, or even earlier, offers further insights into the contribution of progeny nuclides. For instance, Figure 3B shows no peaks at shorter retention times, suggesting that the observed activity did not originate from Ac-225 decay but was already present in the solution before labeling. Accordingly, the appearance of Ac-225 at lower retention times can be confirmed. Subsequently, the fractions were analyzed using gamma spectroscopy by quantifying the proportion of the Francium-221 line at 218 keV immediately after separation and again after 24 h. These indirect measurements of Ac-225, by the gamma lines of its daughters Fr-211 and Bi-213, either by TLC or HPLC, are typical workarounds for quantification of Ac-225, as it has no direct detectable gamma signal. In this context, the state of the equilibrium may influence the result of the quantification. Therefore, definite time points for measurements and interpretation were chosen to guarantee comparability. However, we found that time points between 1 h and 24 h after disturbance of the equilibrium only had a low impact on the obtained values.

An example of one analytical radio-HPLC chromatogram is given in Figure 3, demonstrating an RCP of ≥97%. Purity was calculated by determining the area under the peaks. The average RCC of the validation cycles was 98.0 ± 1.1%, and the average RCY was 86.7 ± 2.7%, indicating a reliable process that can be operated within the desired parameters. Differences occurring between RCC and RCY occur from process steps in general, i.e., vial transfer, vial retention, or sterile filtration.

During the subsequent routine productions of [^225^Ac]Ac-DOTA-LM3, a mean RCC of 98.3 ± 1.5% at the end of synthesis was observed (*n* = 19). After successful validation, radio-HPLC runs were not performed by default for batch release due to the long duration of up to 24 h for measuring the decayed samples and because of the unlikelihood of a significantly decreased RCP. However, after changing peptide suppliers or using new batches of chemicals, a revalidation is recommended to ensure product safety. Aside from that, as a source of information, TLC was considered reliably accurate. The synthesis yielded activities of the final drug product between 12 MBq and 46 MBq (decay-corrected RCY = 89.6 ± 6.7%; productions were used for as many as 7 patients). Further quality control parameters included dose activity, visual control, pH, and spectroscopy for nuclide identification, radiochemical conversion, intermittently RCP (radio-HPLC), sterile filter integrity (bubble point), and endotoxin test. An overview of specifications and quality control results is given in Table 1. All values were within the specified range and provided a drug product of adequate quality.

### 2.2. In Vitro Stability

The stability of the drug product was investigated without further processing during the validation process by testing the pure formulation and incubation of an aliquot in human serum at 37 °C (see Figure 2B). For the latter, a concentration of 100 kBq/mL was used, whereas the pure formulation had a concentration of 1 MBq/mL. Analysis was performed using radio-TLC with a TLC-decay time of 1 h. The investigation over 22 days showed that the compound does not release more than 10% of free Actinium-225 over the first five days, making it suitable for human injections. Afterwards, a substantial release was observed, especially in the highly concentrated pure formulation. However, the TLC results can only indicate the release of the radiometal and do not provide any insight into the molecules’ inherent chemical and metabolic stability. Nevertheless, due to the low activities after dilution, no stability investigations using HPLC were carried out.

### 2.3. Radio-HPLC

Direct HPLC measurements of Actinium-225 using conventional detectors are very challenging due to the low activities used and the occurrence and subsequent detection of the daughter nuclides. To overcome both problems, indirect detection combining HPLC fraction collection and subsequent gamma spectroscopy has become the method of choice [33,34]. Therefore, lower levels of activity can be quantified, and serial measurements 1 to 24 h after collection resolve the challenges caused by the progenies. Figure 3 provides an overview of the fractional separation of [^225^Ac]Ac-DOTA-LM3. In this example, sixteen fractions were automatically collected in intervals of 30 s and subsequently analyzed in a gamma spectrometer. Figure 3A shows the quantification of the vials right after measuring and analyzing the gamma line of Francium-221 at 218 keV. Notably, detection of Francium-211 results in a higher signal intensity compared to Bismuth-213. Two peaks can be identified in the chromatogram: a minor at 2.5 min (18.5%) and a major peak at 9.5 min (75.3%). The minor peak refers to the free isotopes, and the largest peak to the corresponding product. Furthermore, an additional small third peak could be identified (t_r_ = 12.5 min, 5.9%); however, it cannot be delineated in the chromatogram presented in Figure 3A. When measuring the same vials again after 24 h, the peak at 3.0 min completely disappeared in the chromatogram (Figure 3B), indicating that this peak was only generated by shorter living isotopes like Fr-221 and Bi-213 that were present in the labeling mixture from the beginning, and that these fractions did not contain any Actinium-225 directly after separation. Otherwise, Ac-225 would permanently produce a Fr-221 and Bi-213 gamma line. Therefore, the calculated purity is 97.2% for the desired product and 0.7% for the free isotopes. Detailed evaluation of the chromatogram (Figure 3C) also visualizes the third peak at t_r_ = 12.5 min (2.1%), which is also in correspondence with an uncharacterized impurity observed during the labeling with Lutetium-177 and Gallium-68. It shows a slightly longer retention time than the desired product [^225^Ac]Ac-DOTA-LM3. Remarkably, comparisons using identical methods present similar retention times for [^177^Lu]Lu-DOTA-LM3 (t_r_ = 7.5 min) and [^68^Ga]Ga-DOTA-LM3 (t_r_ = 7.0 min) (see Supplementary Materials). Overall, this analytical method indicates good RCP for the labeling of DOTA-LM3 with Actinium-225.

### 2.4. Patient Example

Two cases of patients treated in a TANDEM PRRT with ^177^Lu/^225^Ac-labelled DOTA-LM3 have already been reported by Speicher et al. and Perrone et al. [35,36]. In this work, we also report a case of a patient with a well-differentiated, highly proliferative, non-functional neuroendocrine pancreatic neoplasm with lymph nodular involvement, voluminous hepatic and multiple pulmonary filiae as well as excessive osseous metastasis. The patient underwent stand-alone therapy with [^225^Ac]Ac-DOTA-LM3 after progressing under 3 cycles of [^177^Lu]Lu-DOTA-TATE. The cumulative administered activity was 23.8 GBq of Lutetium-177 and 16 MBq of Actinium-225. The patient received two cycles of [^225^Ac]Ac-DOTA-LM3 with an interval of 11 months and showed partial remission (THERCIST) after these two cycles without clinical or significant side effects on bone marrow, liver, or renal function.

## 3. Materials and Methods

### 3.1. Chemical

The DOTA-LM3 precursor was bought in research-grade quality (purity > 99%) from piCHEM (Grambach, Austria). Actinium-225 for radiolabeling was obtained from ITM (Munich, Germany) in the form of [^225^Ac]AcCl_3_ in HCL (0.1 M) in a V-shaped vial. No further purification was conducted before the use of Actinium-225. The pH was controlled at the start and end of labeling using pH stripes (Merck, Darmstadt, Germany; pH 2–9).

Stock solutions prepared with TraceSelect-water^®^ (Sigma-Aldrich, St. Louis, MO, USA) were used for labeling. All chemicals were of the highest chemical grade available. Solvents for HPLC were obtained as HPLC grade from Merck (Germany). The test for bacterial endotoxins was performed using a Nextgen PTS (Charles River, MA, USA) device with suitable single-use consumables. Radioactivity of the drug products was determined using an ISOMED2010 (NUVIA Instruments, Dülmen, Germany) dose calibrator calibrated for Actinium-225 with sources from ITM by NUVIA Instruments.

Radiolabeling was performed using 2.0 mL of 0.10 M ascorbic acid buffer at pH 4.5 and 25 µg precursor per MBq of Actinium-225 at 95 °C. No further purification of the product was required, and the formulation was performed by dilution with 100 mg sodium ascorbate dissolved in 9 mL of saline and 1 mL of ethanol and subsequent passage through a sterile filter (Sterifix^®^, B. Braun SE, Melsungen, Germany).

Analytical radio-HPLC was performed using an HPLC system (Agilent, Infinity 1200 [34] 100 µL injection loop, 20 µL injection for quality control) equipped with a Ramona* radioactivity detector (Elysia-Raytest, Liège, Belgium), an external BGO scintillator flow cell (300 µL, Elysia-Raytest, Liège, Belgium), and an automated fraction collector. The following column was used: VDSpher PUR 150 C18-E 5 μm, 100 × 4.0 mm VDS Optilab, Berlin, Germany. The mobile phases were acetonitrile (A) and water (B), each containing 0.1% TFA. The gradient with a flow rate of 1.0 mL/min started at 5% of solvent A and increased to 95% of A within 10 min. Fraction volumes were 0.5 mL, resulting in a total of 16 vials. TLCs were analyzed with a miniGita TLC scanner (Elysia-Raytest, Liège, Belgium) and the analysis software Gina 6.3 (Elysia-Raytest, Liège, Belgium) for data interpretation or a Dürer Medical scanner with AIDA software analyzer 5.1. The citrate buffer was prepared by dissolving 2.1 g of sodium citrate (NaCit) and 1.7 g of citric acid (HCit) in TraceSelect water and adjusting the final volume to 50 mL. If not stated differently, the times between the development of TLCs and measurements were1 h.

For gamma spectroscopy, either a high-resolution gamma spectrometer with an HPGe detector or an ISOMED 2151 (Nuvia Instruments) with a NaI scintillation detector was used. Stability studies were performed in human serum (HS) or from unprocessed samples derived from the formulated final product and conducted in triplicate at 37 °C. HS (human male AB plasma, USA origin) was obtained from Sigma-Aldrich, United States. The final procedure used 100 μL of the formulated product solution (100 kBq) added to 1 mL of HS. Radio-TLC was carried out under the analysis conditions previously mentioned.

### 3.2. Clinical Results

Medical use was in accordance with §13 2b, German Medicinal Products Act. All patients had histologically confirmed malignancies and received prior oncologic treatments, including RLTs in some cases. Additional criteria for patient treatment with TAT were willingness to give informed consent, sufficient general condition and renal function, and no known allergies to any of the excipients.

The 57-year-old male patient received two cycles of therapy with [^225^Ac]Ac-DOTA-LM3 following tumor progression after three cycles of PRRT with Lu-177 DOTATOC (total cumulative dose 21.8 GBq). During both cycles of the treatment, he was injected with 8 MBq of [^225^Ac]Ac-DOTA-LM3 in 10 mL saline solution within a period of 1 min between 1 and 4 h after production of the radiopharmaceutical. The second cycle was performed after an interval of 8 weeks. During the second cycle, the patient also received 2 GBq of [^177^Lu]Lu-DOTA-LM3 for subsequent SPECT imaging after therapy. Before and after therapy, PET/CT scans (Biograph Vision 600 Edge, Siemens Healthineers, Forchheim, Germany) with [^68^Ga]Ga-DOTA-LM3 were acquired from the vertex to the proximal femora after 70 min post-injection. A low-dose CT scan (130 keV, 30 mAs, CareDose; reconstructed with a soft-tissue kernel to a slice thickness of 5 mm) was used for attenuation correction and anatomical mapping of the tracer. For the imaging of [^177^Lu]Lu-DOTA-LM3, SPECT/CT images were acquired 24 h after injection with a Siemens Symbia SPECT/CT (Siemens Healthineers, Forchheim, Germany).

After the two cycles of therapy with [^225^Ac]Ac-DOTA-LM3, no hematotoxicity was observed; moreover, the patient demonstrated an increase in hemoglobin levels despite a prior need for regular blood transfusions. Renal function remained stable, and a marked improvement in hepatic transaminases was noted. 

Clinically, there was a pronounced and significant improvement in the patient’s general condition. The patient, who had initially suffered from severe cancer cachexia (extreme loss in weight and muscle mass) and ascites (accumulation of fluid in the abdominal cavity), experienced complete resolution of these conditions by the time of the last [^68^Ga]Ga-DOTA-LM3. It is particularly noteworthy that the patient, who had been bedridden at the beginning of treatment, was able to resume his medical practice, working up to seven hours per day within six months of therapy initiation. Although the patient showed a high response in the numerous bone metastases, this is unlikely to be due to deposition of free Ac-225 in bone. This is supported by our stability studies, which show that the Ac-225-DOTA-LM3 complex undergoes only minimal decomplexation over 20 days in human serum (Figure 2B).

**Figure 4:**
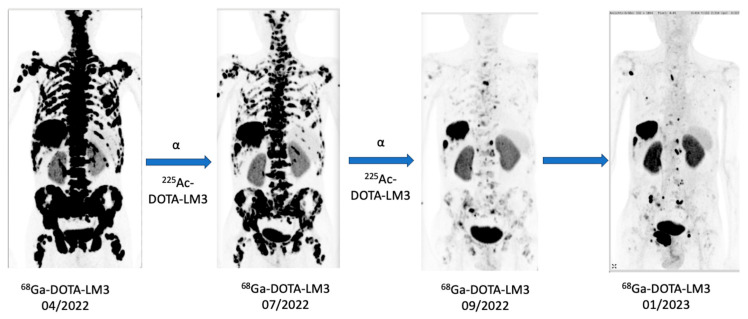
Clinical application of [^225^Ac]Ac-DOTA-LM3 in a NET patient with progressive disease under agonistic [^177^Lu]Lu-DOTA-TATE therapy. Sequential PET/CT images as maximum intensity projections before and after treatment with 8 MBq [^225^Ac]Ac-DOTA-LM3 and 8 MBq Ac-225. In the second cycle, the patient was also injected with 2 GBq of [^177^Lu]Lu-DOTA-LM3 mainly for subsequent single-photon emission computed tomography (SPECT) imaging. The images demonstrate a significant reduction in tumor burden.

Therefore, a significant release of Ac-225 and a primary bone-targeted palliative effect by free radionuclides is unlikely in this case. Minor contributions from released daughter nuclides cannot be completely excluded; however, these observations are intended solely to describe the documented clinical course, and mechanistic conclusions are beyond the scope of this study (Figure 4).

## 4. Conclusions

This study highlights the potential of [^225^Ac]Ac-DOTA-LM3 as a promising radiopharmaceutical for TAT in the treatment course of NET patients. The successful radiolabeling process achieves a high RCP of over 97%, as determined by TLC and HPLC, along with excellent product stability. This ensures a more reliable production process and facilitates the routine use of the compound in clinical practice. We continue to work on more reliable methods for direct detection of the products labeled with different isotopes to present even more accurate measurements in the future. Furthermore, the clinical application in a NET patient who was refractory to therapy with [^177^Lu]Lu-DOTA-TATE showed an encouraging therapeutic outcome, suggesting that Actinium-225-labeled SSTR2 antagonists might provide a valuable alternative for patients unresponsive to therapy with [^177^Lu]Lu-DOTA-TATE or even [^177^Lu]Lu-DOTA-LM3. However, these claims need to be further investigated in head-to-head comparisons and competitive clinical trials and are not intended to be proven by this paper.

Despite these promising results, further clinical data are necessary to validate the general efficacy and safety of [^225^Ac]Ac-DOTA-LM3. Long-term toxicity, dosimetry considerations, and optimization of treatment protocols remain critical aspects for future research. By addressing these questions, therapies with Actinium-225-labeled antagonists could significantly enrich the treatment landscape for NETs and provide a new option for patients with advanced disease stages.

## Figures and Tables

**Table 1:** Summary of release specifications for [^225^Ac]Ac-DOTA-LM3. TLC was performed with citric acid buffer as the mobile phase and with a TLC plate decay time of 1 h.

[^225^Ac]Ac-DOTA-LM3	Specifications	
>97%	>95%	RCP by radio-HPLC
>99%	>95%	RCP by radio-TLC
5.7 ± 0.3	3.5–7.5	pH
complies	colorless	Color
<0.01%	<0.01%	Radionuclide impurity
<5.0 EU/mL	<17.5 EU/mL	Endotoxin
>2500 mbar	>2500 mbar	Bubble point

## Data Availability

The data presented in this study are available in the article. The raw data supporting the conclusions of this article will be made available by the authors upon request.

## Supplementary Materials

The following supporting information can be downloaded at: https://www.mdpi.com/article/10.3390/ph19010172/s1, Figure S1: Radio-HPLC analysis of [^68^Ga]Ga-DOTA-LM3; Figure S2: Radio-HPLC analysis of [^177^Lu]Lu-DOTA-LM3.
