# Supplementary material for: Alpha Therapy Beyond TOC and TATE—Production, Quality Control, and In-Human Results for the SSTR2 Antagonist DOTA-LM3
Source: Pharmaceuticals (Basel). 2026 Jan 19;19(1):172. doi: 10.3390/ph19010172 (PMC12845222; doi:10.3390/ph19010172)
Supplement: Supplementary file 1 [file pharmaceuticals-19-00172-s001.zip › pharmaceuticals-3897295-supplementary.pdf]

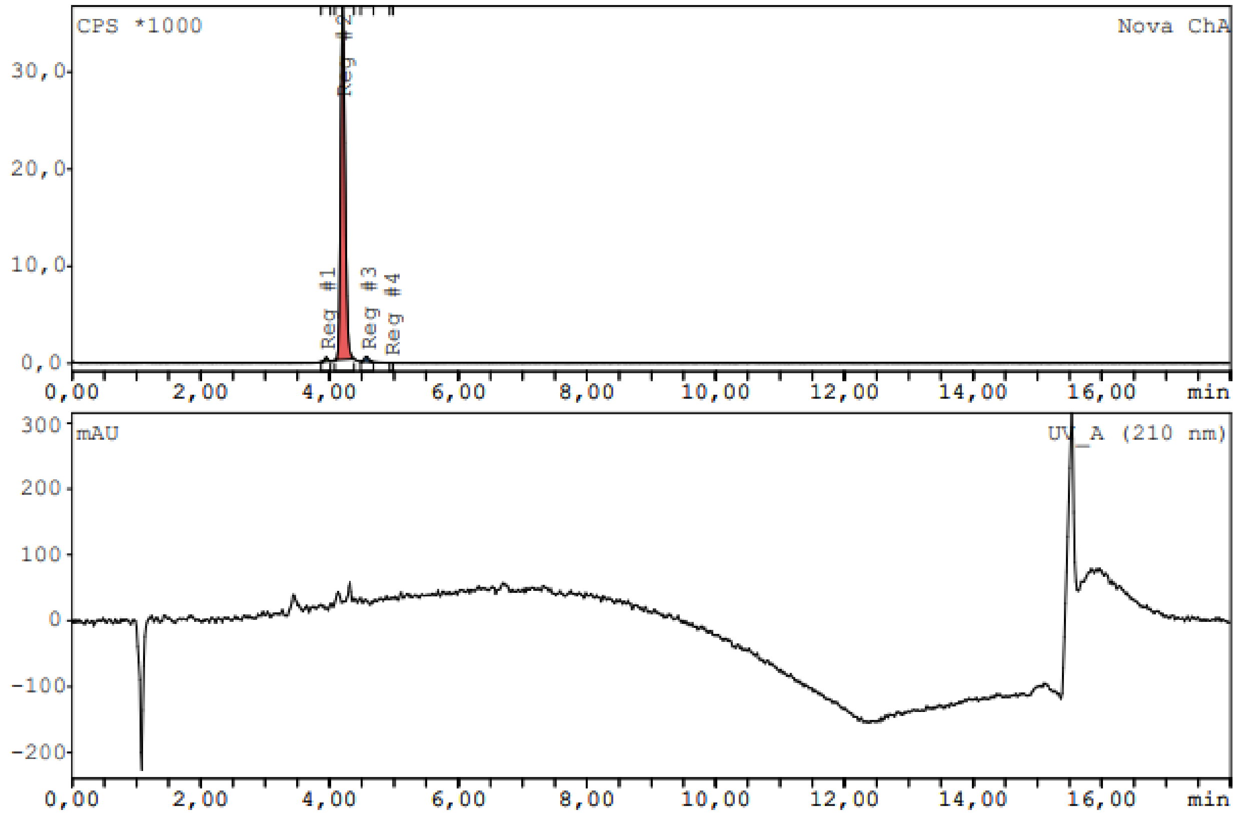


**Probenbeschreibung**

Studie: sugar1
Messung: GaDLM3 5 zu 95, Injektion : 04.06.2025 08:47
Methode: 5TO95 IN 10 von: 12.10.2022 11:04: Position: 16
Stationsnummer: 10
Inj.-Volumen (µl): 40,0
Radioaktivitätsdetektor: Elysia-raytest Gabi Nova / Serien Nr..: 31298
Detector S/N: 0 / Detector Firmware: 0,00 / Detector Scint. type: 0 ()
Software Version: 6.1, Service Pack 2, Erzeugen 6390

**Integration Nova ChA**

| Substanz | R/T min | Typ | Fläche Counts | %Fläche % |
|---|---|---|---|---|
| Reg #1 | 3,95 | BB(M) | 1323,0 | 0,79 |
| Reg #2 | 4,20 | BB(M) | 163809,0 | 97,89 |
| Reg #3 | 4,58 | BB(M) | 2193,0 | 1,31 |
| Reg #4 | 4,95 | BB(M) | 18,0 | 0,01 |
| Summe in ROI | - | - | 167343,0 | 100,00 |

*Figure S1: Radio-HPLC analysis of [$^{68}$Ga]Ga-DOTA-LM3.*

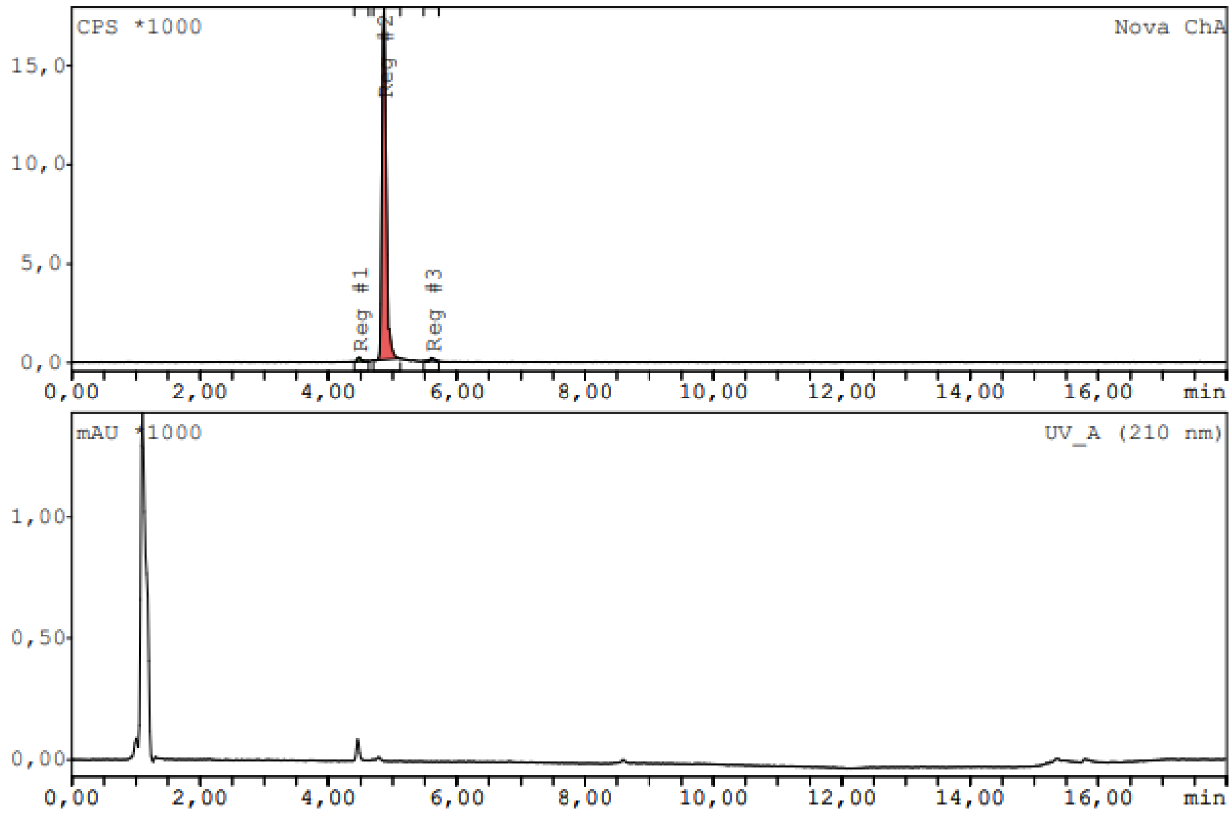


**Probenbeschreibung**

| | | | | | |
|---|---|---|---|---|---|
| Studie: | sugar1 | | | | |
| Messung: | LuDLM3 5 zu 95, Injektion : | | 04.06.2025 15:53 | | |
| Methode: | 5TO95 IN 10 | von: | 12.10.2022 11:04: | Position: | 82 |
| Stationsnummer: | 10 | | | | |
| Inj.-Volumen (µl): | 20,0 | | | | |

Radioaktivitätsdetektor: Elysia-raytest Gabi Nova / Serien Nr..: 31298
Detector S/N: 0 / Detector Firmware: 0,00 / Detector Scint. type: 0 ()
Software Version: 6.1, Service Pack 2, Erzeugen 6390

**Integration Nova ChA**

| Substanz | R/T min | Typ | Fläche Counts | %Fläche % |
|---|---|---|---|---|
| Reg #1 | 4,48 | BB(M) | 801,00 | 0,97 |
| Reg #2 | 4,87 | BB(M) | 80934,17 | 98,17 |
| Reg #3 | 5,62 | BB(M) | 709,33 | 0,86 |
| Summe in ROI | - | - | 82444,50 | 100,00 |

*Figure S2: Radio-HPLC analysis of [$^{177}$Lu]Lu-DOTA-LM3.*
